# ^210^Pb-^226^Ra disequilibria in young gas-laden magmas

**DOI:** 10.1038/srep45186

**Published:** 2017-03-24

**Authors:** Mark Reagan, Simon Turner, Heather Handley, Michael Turner, Christoph Beier, John Caulfield, David Peate

**Affiliations:** 1 Department of Earth and Environmental Sciences, University of Iowa, Iowa City, Iowa, IA 52242, USA; 2 Department of Earth and Planetary Sciences, Macquarie University, NSW 2109, Australia; 3 GeoZentrum Nordbayern, Friedrich-Alexander Universität Erlangen-Nürnberg, Schlossgarten 5, D-91054 Erlangen, Germany; 4 Trinity College Dublin, School of Natural Sciences, Department of Geology, Dublin 2, Ireland

## Abstract

We present new ^238^U-^230^Th-^226^Ra-^210^Pb and supporting data for young lavas from southwest Pacific island arcs, Eyjafjallajökull, Iceland, and Terceira, Azores. The arc lavas have significant ^238^U and ^226^Ra excesses, whereas those from the ocean islands have moderate ^230^Th and ^226^Ra excesses, reflecting mantle melting in the presence of a water-rich fluid in the former and mantle melting by decompression in the latter. Differentiation to erupted compositions in both settings appears to have taken no longer than a few millennia. Variations in the (^210^Pb/^226^Ra)_0_ values in all settings largely result from degassing processes rather than mineral-melt partitioning. Like most other ocean island basalts, the Terceira basalt has a ^210^Pb deficit, which we attribute to ~8.5 years of steady ^222^Rn loss to a CO_2_-rich volatile phase while it traversed the crust. Lavas erupted from water-laden magma systems, including those investigated here, commonly have near equilibrium (^210^Pb/^226^Ra)_0_ values. Maintaining these equilibrium values requires minimal persistent loss or accumulation of ^222^Rn in a gas phase. We infer that degassing during decompression of water-saturated magmas either causes these magmas to crystallize and stall in reservoirs where they reside under conditions of near stasis, or to quickly rise towards the surface and erupt.

Disequilibrium between uranium-series nuclides in lavas, which is created by partitioning of elements between mineral, melt, fluid, and vapor phases and diminishes by radioactive decay, provides unique information about the timescales and processes of magma generation, transport, and differentiation^1^,^2^,^3^,^4^,^5^. In terms of just how rapid some of these processes might be, many lavas exhibit disequilibria between ^210^Pb (T_1/2_ = 22.6 y), ^210^Po (T_1/2_ = 138 days), and ^226^Ra (T_1/2_ = 1,600 y), illustrating that magmatic differentiation processes can persist nearly to eruption^6^,^7^,^8^,^9^.

Activities of ^210^Pb (indicated by parentheses) are commonly less than those of its long-lived parent (^226^Ra) in ocean island basalts (median (^210^Pb/^226^Ra) = 0.76)^10^ and in some spreading centers^3^. These ^210^Pb deficits commonly have been attributed to Pb-Ra fractionation during melting and rapid transport to the surface^3^,^10^. In contrast, the median (^210^Pb/^226^Ra) for arc lavas is 1.0^10^, although values range widely, which is typically attributed to Rn loss or gain in the decades leading to eruption^5^,^7^,^10^,^11^,^12^,^13^,^14^. We explore the hypothesis that ^210^Pb-^226^Ra disequilibrium generated by mantle melting generally decays during the time needed for magmas to migrate to the surface in all tectonic settings, and most measured disequilibrium reflects open-system Rn degassing and accumulation. The prevalence of near equal (^210^Pb) and (^226^Ra) values in volatile-rich arc magmas, but ^210^Pb deficits in less-volatile-rich ocean island lavas typically seems paradoxical. Nevertheless, we contend that it is a natural result of the differences in the relative abundances of volatile species and in the modalities of degassing for different tectonic settings.

To explore the origin of ^210^Pb-^226^Ra disequilibria in magmas, we analyzed young volcanic rocks from ocean islands and arcs. The requirement for including samples in this study is that they erupted less than a few decades before the date of measurement. The data were acquired over a period of several years (2008–2015) as suitable samples became available. Most of the analyzed samples are from locations where ^210^Pb data are rare, or represent lava types that erupt infrequently. Several of our samples recently erupted from the Tonga, Kermadec, and Vanuatu arcs in the southwest Pacific, including the Earth’s youngest rhyolite. We also analyzed a “lava balloon” erupted in 1999 offshore of Terceira, providing the first ^210^Pb data from the Azores. Finally, a sample from the explosive 2010 eruption of Eyjafjallajökull in Iceland was included to investigate ^210^Pb-^226^Ra disequilibria in tephras deposited after an eruption that was responsible for extensive disruption to air traffic.

## Recent eruptive products

With one exception, all of the samples studied here erupted within 15 years of the analysis date (Table 1). Most samples included in this study are crystal-poor to aphyric. Several of these samples are from the Tonga arc. Samples HH09-01 and -02 are two separate scoria clasts collected during the eruption of Hunga Ha’apai in April 2009. Phenocrysts are rare in these samples, with <1% augite and plagioclase, with trace orthopyroxene in a vesicular matrix consisting of brown glass choked with laths of plagioclase and augite. HR06 is pumice collected from the November 2006 eruption of Home Reef, a near-surface volcanic center 25 km SSW of Late volcano. This sample has about 2% phenocrysts of augite and plagioclase, which form rare glomerocrysts with magnetite. The matrix is highly vesicular colorless glass. Sample F0805 is a crystal poor, vesicular dacite from the summit of Fonualei volcano that is inferred to have erupted between 1979 and 1990^15^.

Our sample of rhyolite from Havre volcano erupted in July 2012 just west of the Kermadec volcanic front at about 1000 meters water depth. The volume of the eruption is estimated at about 1.5 km^3^, making it the largest and deepest recorded silicic submarine eruption^16^. A pumice raft resulting from this eruption spread across the southwest Pacific and Tasman Sea over the ensuing months^17^. Our sample is a spherical homogenous light gray pumice clast of about 900 cm^3^ collected from a high-tide accumulation of Havre pumice on a Sydney Harbour beach in February 2014. It consists of frothy fresh glass with about 1% plagioclase and trace augite.

Van A1 is a vesicular lava bomb with abundant mm-scale plagioclase collected shortly after eruption from Yasur volcano, Vanuatu in August 2008^18^.

The Serreta sample is a glassy quenched margin of one of the gas-inflated basaltic pillows that breached the sea surface off of Terceira island in the Azores in February 1999^19^.

Finally, sample EJ-1 is ash deposited during the April 15, 2010 eruption of Eyjafjallajökull in Iceland. It is a portion of sample 2 from Gislason *et al*.^20^, where it is described in detail. Sigmarsson *et al*.^21^,^22^ analyzed a separate fraction of this sample. All tephras from this eruption are more than 85% juvenile^23^. The benmoreite ash is glassy and relatively crystalline, with abundant bimodally zoned crystals of olivine, plagioclase, augite, and magnetite^24^.

We undertook full compositional characterization of these samples (i.e. major and trace elements, Sr-Nd isotopes and ^238^U-^230^Th-^226^Ra-^210^Pb disequilibria; Table 1) to distinguish between origins of (^210^Pb/^226^Ra) disequilibria by magma generation and differentiation in different tectonic settings and for different magma types.

## Results

**Figure 1:**
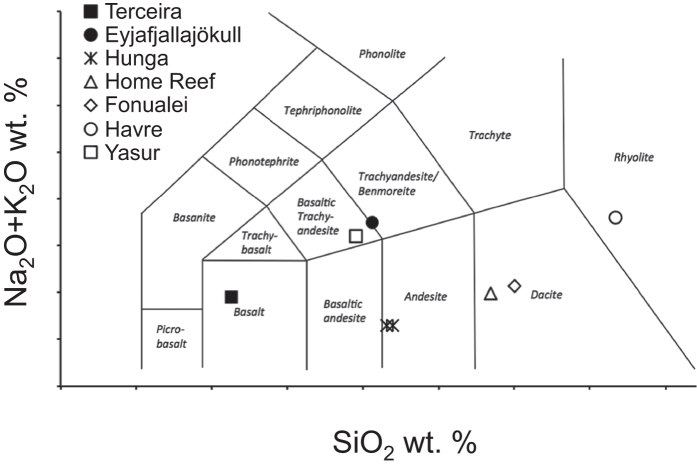
Plot of total alkalis versus silica illustrating the bulk composition of the island arc ([open symbols]Vanuatu: Van A1; Tonga: HH09-01, HH09-02, HR06, and F0805; and Kermadec: PC) and ocean island ([closed symbols] Azores: Serreta; Iceland: EJ-1) samples analyzed here. Data are from Table 1.

With the exception of the Serreta sample from the Azores, all of the samples analyzed here have <3.6 wt.% MgO and <50 ppm Cr indicating that they are significantly differentiated from their respective mantle-derived parental magmas (Table 1). The sample from Yasur, Vanuatu is a basaltic trachyandesite (Fig. 1) with a bulk-rock major and trace element composition similar to those of other lavas erupted from this steady-state volcano^25^,^26^. Tonga samples from Hunga are low-K andesites with SiO_2_ between 57.5 and 58 wt.%. Tonga samples from Home Reef and Fonualei samples are low- and medium-K dacites with 64.5 to 66 wt.% SiO_2_ respectively. The glass in our sample of the Havre, Kermadec pumice is a medium-K rhyolite with 73 wt.% SiO_2_ and an overall composition matching those of other pumice glass from this eruption^27^ (Table 1). Our ocean island sample from Eyjafjallajökull, Iceland is a benmoreite with 57 wt.% SiO_2_ and combined concentrations of Na_2_O and K_2_O equal to 7 wt.%. The Serreta sample from Terceira, Azores is relatively primitive alkali basalt with 8.57 wt.% MgO, 235 ppm Cr, 47 wt.% SiO_2_ and 3.8 wt.% total alkalis.

**Figure 2:**
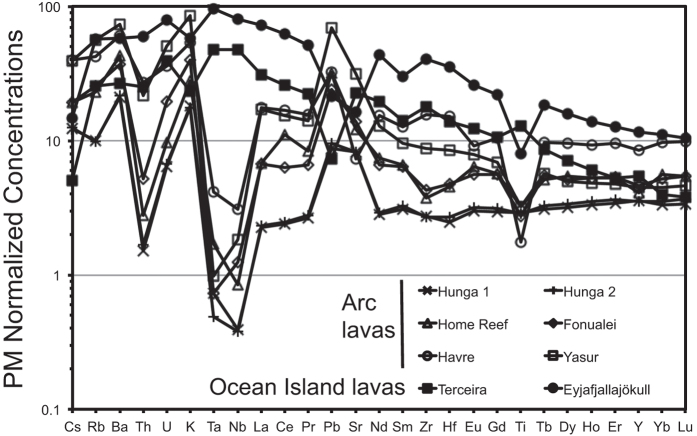
Primitive mantle-normalized incompatible element concentrations for samples listed in Table 1 from the island arcs and ocean islands illustrating the wide-ranging compositions analyzed here. See Fig. 1 and the text for sample locations.

All of the samples from western Pacific volcanic arcs (Tonga, Kermadec, Vanuatu) are enriched in fluid mobile elements such as Cs, Rb, Ba, U, K, Pb, and Sr and depleted in Nb and Ta with respect to light rare-earth elements (Table 1; Fig. 2). Th/La ratios for samples from the Tonga arc are in the range of those recorded for mid-ocean ridge basalts, whereas Th/La values are higher for the lavas from Yasur and Havre volcanoes. The two ocean island samples have relatively smooth incompatible element patterns with steep rare earth element slopes and negative K and Pb anomalies. The more evolved samples from both settings have negative Sr and Ti anomalies because of significant plagioclase and titanomagnetite fractionation.

With the exception of the Havre rhyolite, the ^143^Nd/^144^Nd radiogenic isotope data for the analyzed samples vary within a restricted range from 0.513030 to 0.512925 (Table 2), whereas the arc samples are offset to higher ^87^Sr/^86^Sr ratios (0.703540 to 0.703769) compared to the ocean island samples (0.703257 to 0.703533). The Havre rhyolite has a greater offset, with relatively high ^143^Nd/^144^Nd and ^87^Sr/^86^Sr ratios (0.513115, 0.703705). Thorium isotopic compositions of the arc samples vary from 1.70 to 0.93, in line with regional variations^28^,^29^. Similarly, the (^230^Th/^232^Th) ratio in the benmoreite from Eyjafjallajökull is comparable to those of other silicic Icelandic lavas^22^. In contrast, the Serreta basalt has (^230^Th/^232^Th) = 1.40, which is the highest ratio thus far measured for lavas from the Azores^30^,^31^.

**Figure 3:**
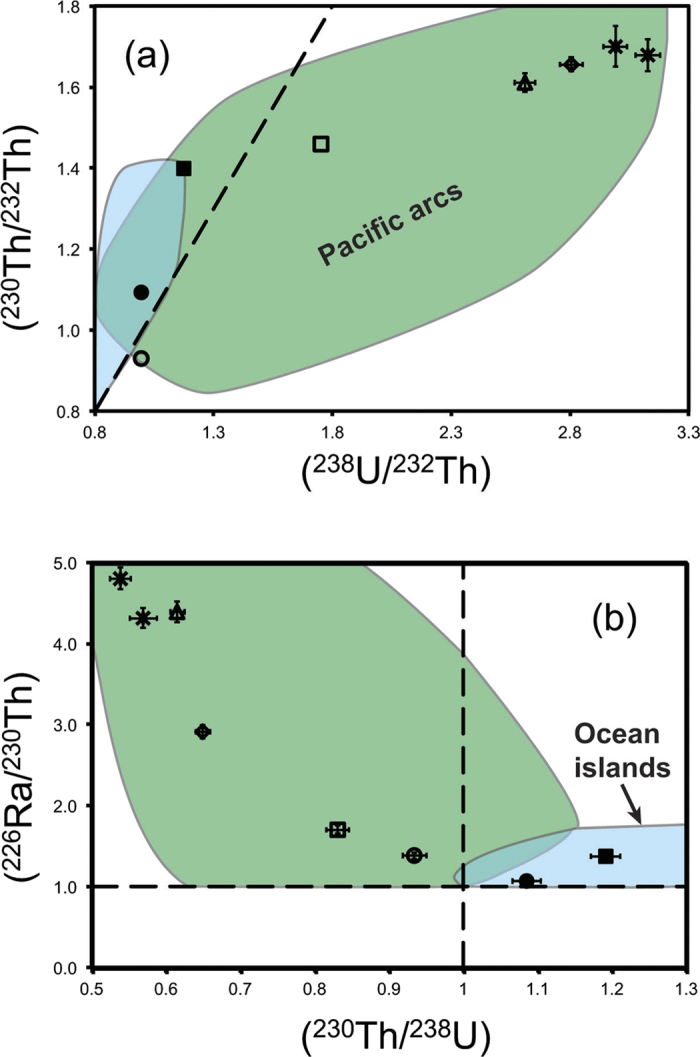
Plots of (**a**) (^230^Th/^232^Th) versus (^238^U/^232^Th) and (**b**) (^226^Ra/^230^Th) versus (^230^Th/^238^U) for samples listed in Table 2 from island arcs and ocean islands. The blue and green fields in (**a**) and (**b**) mark the range of ocean island basalts and western Pacific island arcs respectively^2^,^12^,^15^,^29^,^32^,^33^,^34^,^35^,^36^ for comparison. Dashed lines denote secular equilibrium. Symbols as in Fig. 1. Error bars are illustrated for values larger than the sample symbols.

Uranium, Th, and Ra concentrations for the samples analyzed here vary over wide ranges (0.13 to 1.68 ppm, 0.13 to 5.05 ppm, and 116 to 654 fg/g respectively; Table 2). Uranium isotope ratios are within error of secular equilibrium for most samples. The exception is the Fonualei dacite with (^234^U/^238^U) = 1.016, which is attributed to seawater alteration. The arc samples have significant ^238^U and ^226^Ra excesses over ^230^Th, with (^230^Th/^238^U) ranging from 0.54 to 0.93 and (^226^Ra/^230^Th) from 1.38 to 4.80 (Fig. 3). The Havre rhyolite has the least U-Th-Ra disequilibria of these samples. Sample Van A1 from Yasur also has relatively low (^238^U/^230^Th) and (^230^Th/^232^Th) values (0.83 and 1.46 respectively). These values are similar to those reported for lavas erupted from Yasur in 1975 and 1993^29^.

In contrast with the arc lavas, but like ocean island basalts in general, the Serreta basalt has ^230^Th and ^226^Ra excesses ((^230^Th/^238^U) = 1.19 and (^226^Ra/^230^Th) = 1.37). Excesses of ^230^Th and ^226^Ra are less pronounced in the relatively fractionated benmoreite from Eyjafjallajökull ((^230^Th/^238^U) = 1.09 and (^226^Ra/^230^Th) = 1.07).

Measured ^210^Po activities in leached samples range from 0.10 to 1.4 dpm/g (see Supplementary Information Table S1). This contrasts with the 3 to 14 fold higher activities in the leachates of the youngest samples (1.5 to 6.5 dpm/g) resulting from significant Po in sublimates adhered to vesicle walls and ash particles during eruption^37^. The inferred (^210^Pb/^226^Ra)_0_ ratios of leached samples are all within analytical error of secular equilibrium with the exception of a 14% ^210^Pb excess in the Home Reef sample and a significant ^210^Pb deficit in the Serreta sample ((^210^Pb/^226^Ra)_0_ = 0.71; Table 2).

Our (^210^Pb/^226^Ra)_0_ data for Iceland sample EJ-1 is within analytical error of the value published in Sigmarsson *et al*.^22^, although our measured concentrations of U, Th and Ra are lower by 6.5–15%, suggesting our fraction of the bulk tephra sample had higher proportions of mineral particles versus glass.

## Discussion

The data for young samples presented above are discussed in terms of geographical area and tectonic setting below. We go on to compare these data to the global dataset and address wider implications of short-lived radionuclide disequilibria in lavas.

### Southwest Pacific arc samples

**Figure 4:**
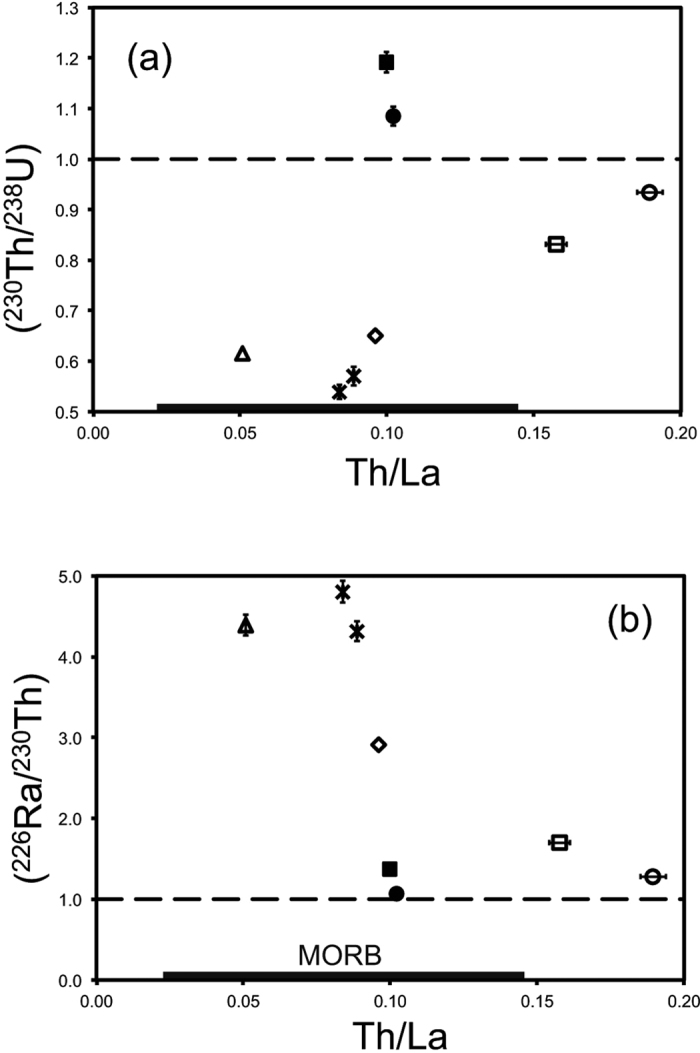
Plots of (**a**) (^230^Th/^238^U) and (**b**) (^226^Ra/^230^Th) against Th/La for samples listed in Table 2. The black bars represent the range of Th/La in MORB glasses^42^. For samples from western Pacific arcs, the low (^230^Th/^238^U) and high (^226^Ra/^230^Th) values associated with samples with high Th/La suggests that these ratios largely reflect the degree to which Th is mobilized from the subducting plate. Symbols as in Fig. 1. Error bars are illustrated for values larger than the sample symbols.

The samples from Hunga, Home Reef, and Fonualei in the Tonga arc have large ^238^U and ^226^Ra excesses that are typical for, indeed only found in, oceanic island arcs of the western Pacific^27^,^28^,^31^,^32^. These disequilibria, as well as the trace element patterns and elevated Sr isotope values, are inferred to reflect fluid addition of U, Ra, and Sr to the mantle wedge source from the subducting Pacific plate, in keeping with previous interpretations of U-series data for Tonga arc lavas^28^,^32^. The subducting Pacific plate beneath the Tonga arc is relatively cold^38^ and the high H_2_O/Ce^39^, and low Th/La ratios for these lavas (Fig. 4) imply that fluids released from the Pacific plate are water rich and the subducting plate minimally melts. The Havre rhyolite marks an end-member isotopic composition for the Tonga-Kermadec system, with its unusual pairing of low (^230^Th/^232^Th) and high ^143^Nd^/144^Nd compared to oceanic basalts^34^ and typical subducted lithologies^40^,^41^. These observations are consistent with derivation of most Nd from the local mantle, and most Th from subducting sediment.

The tight negative and positive correlations respectively between (^226^Ra/^230^Th) and (^230^Th/^238^U) versus Th/La for our arc samples (Fig. 4) suggests that much of the variation in ^226^Ra-^230^Th-^238^U disequilibria relates to the degree to which Th is mobilized from the subducting plate rather than bulk assimilation of crust or time-scale of fractionation. The Home Reef dacite and Hunga andesites have similarly high (^226^Ra/^230^Th) ratios, consistent with similar century to millennium time frames of fractionation for these lavas^32^. The smaller ^226^Ra excess but similar U-Th systematics for the Fonualei dacite compared with the other Tonga samples imply a modestly extended time-frame of fractionation, perhaps a few thousand years. The Havre rhyolite has comparatively low ^238^U and ^226^Ra excesses, a low (^230^Th/^232^Th) value, and a comparatively high Th/La value with respect to the Tonga lavas. We attribute all of these observations to a more significant transfer of Th from subducting sediment^28^, which resulted in less initial ^238^U-^232^Th-^226^Ra disequilibria in the parental magmas for the Havre rhyolite compared with the Tonga arc, and a time frame of fractionation that also did not exceed several thousand years. Similarly, the incompatible trace element enriched basaltic trachyandesite from Yasur has moderate excesses of ^238^U and ^226^Ra over ^230^Th compared with the Tonga arc samples, which might also reflect transfer of Th from subducted sediment^29^.

**Figure 5:**
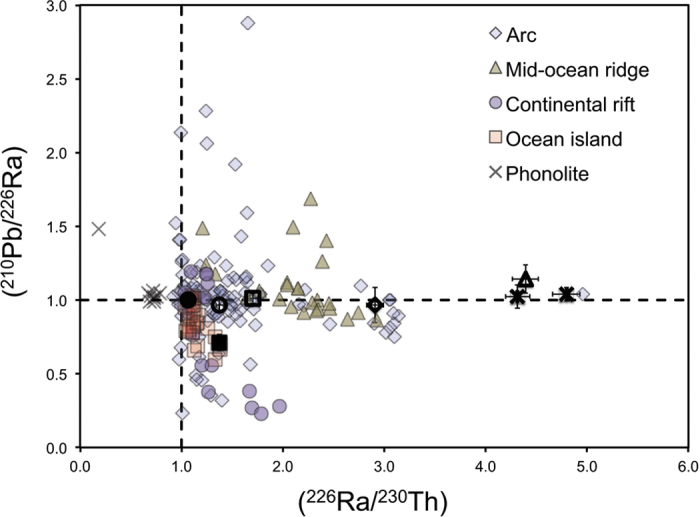
Plot of (^210^Pb/^226^Ra) versus (^226^Ra/^230^Th) showing data from this paper with error bars (island arc samples = black diamonds; ocean arc samples = black squares) and published data for lavas and tephras from a variety of tectonic settings^9^,^10^,^43^,^44^,^45^,^46^. The phonolite whole rock from Tristan da Cunha^8^ and phonolite glass separates from Mount Erebus^9^ are shown separately because they demonstrably have had (^226^Ra) lowered by crystal fractionation of K-rich feldspars or amphiboles. Data were excluded for samples with unusually high Pb concentrations and high (^210^Pb/^226^Ra) values that likely reflect the assimilation of young ^210^Pb-enriched sublimates^5^,^36^,^47^. Dashed lines denote secular equilibrium. Arcs have wide-ranging (^210^Pb/^226^Ra) values but have a median value of equilibrium, whereas ocean island basalts have a median value of 0.76, and rarely have ^210^Pb excesses. We attribute these variations to differences and volatile profiles and modes of degassing in these different tectonic settings. See text for further explanation.

Most lavas from the Tonga-Kermadec and Vanuatu arcs have (^210^Pb/^226^Ra)_0_ ratios within analytical error of 1 (Table 2, Fig. 5). The U-Th-Ra and trace element systematics outlined above indicate that the parental magmas for these lavas were water rich. The equilibrium (^210^Pb/^226^Ra)_0_ values indicate that disequilibrium created by any process including fluid transfer from the subducting plate, melting, and deep degassing decayed away before eruption^9^. Moreover, the last stage of ^222^Rn degassing must have occurred over a time period short enough to be undetectable using ^210^Pb-^226^Ra disequilibria^12^. The small ^210^Pb excess in the Home Reef pumice is can be explained by Rn transfer in a gas phase to the dacite from un-erupted, more mafic recharge magma that may have triggered the eruption^14^,^48^.

### Atlantic Ocean island samples

The Serreta alkaline basalt from Terceira is characterized by an unusually high (^230^Th/^232^Th), but ^230^Th and ^226^Ra excesses that are similar in scale to those generally observed for alkaline ocean island basalts elsewhere, which we interpret to reflect the dynamics of melting garnet-bearing mantle^49^,^50^,^51^. The enrichment of U over Th in the source of this basalt that lead to the high Th isotopic composition, therefore, likely took place more than 350,000 years before magma genesis. This ancient U enrichment could have resulted from mantle carbonation^52^.

The benmoreite we analyzed from Eyjafjallajökull has a major element, trace element, and isotopic composition that is consistent with it representing a mixture between an alkaline basalt and an alkali rhyolite or trachyte^21^,^24^. Its low (^226^Ra/^230^Th) value (1.066) compared to a basalt erupted in 2010 from Fimmvörðuháls on Eyjafjallajökull’s flank (1.368)^22^ is most readily explained by several thousand years of aging during differentiation and storage of the silicic mixing end-member.

The initial (^210^Pb/^226^Ra)_0_ ratio of 0.71 for the Serreta sample is similar to the values observed for most^10^,^34^,^35^ but not all^48^ ocean islands and basalts erupted in continental rift settings^45^,^46^. Basalts from Surtsey, Eldfell, and Fimmvörðuháls in Iceland have comparable ^210^Pb deficits^22^,^46^. The origin of these ^210^Pb deficits is a focus of the next section.

In contrast with Icelandic basalts, the benmoreite from Eyjafjallajökull has a (^210^Pb/^226^Ra)_0_ ratio within error of equilibrium, whereas both the alkaline basalt and trachyte erupted at Eyjafjallajökull have ^210^Pb deficits^22^, and mixing between these particular magmas could not have resulted in the equilibrium (^210^Pb/^226^Ra)_0_ value of the benmoreite. Therefore, like the arc samples discussed above, the benmoreite erupted in 2010 appears to have resided in the crust long enough for any original ^210^Pb-^226^Ra disequilibrium to decay away. These data also suggest that the basalt that triggered the 2010 eruption was different than the one inferred to have mixed to generate the benmoreite (see also ref. 24).

### ^210^Pb in gas-laden magma

Magma migration and degassing can strongly affect the (^210^Pb/^226^Ra) values of magmas because of the volatility of Rn. This volatility is illustrated by the near absence of ^222^Rn in freshly erupted lavas from ocean island and arc settings^53^. In contrast, Pb is only weakly volatile^5^. Thus, to produce measurable ^210^Pb deficits, magmas must continually lose Rn for a minimum of about two years. After a century of degassing, magmas will have (^210^Pb/^226^Ra) values close to zero. Comparatively longer durations of degassing are required to produce ^210^Pb deficits of a particular magnitude if Rn loss is less than 100% efficient^5^ or is discontinuous^14^. Excesses of ^210^Pb over ^226^Ra can be generated relatively rapidly by transfer of Rn-bearing gasses or fluids from greater volumes of magma and into smaller volumes of magma^48^. In this section, we explore whether Rn-Ra fractionation is the principal cause of variations in (^210^Pb/^226^Ra) values for magmas from both ocean island and arc settings.

The relative concentrations of the volatile species CO_2_ and H_2_O in parental magma impact how and when a magma degasses and the consequences of this degassing on its physical properties. Both CO_2_ and H_2_O concentrations in primary ocean-island basalts have been estimated to be from several tenths of a percent up to 1 wt.%^54^,^55^. Volatile concentrations in basalts from the Azores and Iceland appear to be in keeping with these values. Olivine-hosted melt inclusions from the basalt lava balloons erupted in 2001 from Terceira, Azores preserve water contents of up to 0.9 wt.% H_2_O and 1500 ppm CO_2_^19^. Olivine-hosted glass inclusions in basalts from Pico island near Terceira have as much as 0.4 wt.% CO_2_, implying even higher concentrations in parental magmas^52^. Melt inclusions in olivines from basalts erupted from Fimmvörðuháls in 2010, which is a flank vent for Eyjafjallajökull, have up to 1 wt.% water^56^. Olivine-hosted glass+bubble inclusions in basalts erupted from Laki volcano in AD 1783–1784, 130 km to the northwest of Eyjafjallajökull in Iceland, have up to 0.5 wt.% CO_2_^57^.

CO_2_ concentrations of primitive arc basalts, including those of the western Pacific arcs, are poorly known because of extensively degassing. Using global volatile and magma fluxes as guides, CO_2_ concentrations have been estimated to be 0.3 to 1.3 wt.% CO_2_^58^. We presume that CO_2_ concentrations in lavas from the western Pacific arcs are in this range. H_2_O concentrations are significantly higher in primitive arc magmas compared with ocean island basalts, with many mafic lavas, including those for the Tonga arc, recording water concentrations in the 4–6 wt.% range^59^,^60^. Based on gas emissions and compositions of olivine-hosted glass inclusions, parental basaltic magmas for Yasur volcano, Vanuatu have approximately 1–2 wt.% H_2_O and 0.25 wt.% CO_2_^25^.

Primitive basalts with several tenths of a percent CO_2_ in both ocean island and arc settings would typically saturate in a CO_2_-rich vapor phase and potentially begin open-system degassing at deep crust or upper mantle depths^55^,^58^. CO_2_ dominated degassing has little effect on mineral stability and magma viscosity^61^,^62^. H_2_O largely remains dissolved in magmas until the upper several km of crust, where it becomes considerably less soluble^63^. Open-system loss of H_2_O from silicate magmas in the upper crust strongly affects viscosity, because it lowers magma temperatures, enhances polymerization, and causes bubble and mineral growth. Latent heat released by crystallization can mitigate the viscosity increase by raising the temperature of a magma^64^, but overall, the loss of a water-rich volatile phase from magmas typically increases viscosity by orders of magnitude^65^.

Volatile phase saturation also pressurizes the system by bubble growth, creating a driving force towards eruption that is moderated by open system loss of the gas phase^65^. It is the unsteady interplay between changes in viscosity, pressurization, and conduit/reservoir geometry that determines whether water loss will cause a magma to freeze and stall out in the crust, or to migrate rapidly to the surface and erupt^66^,^67^,^68^.

Magmas with relatively low H_2_O/CO_2_ ratios, such as those found in most ocean islands, typically arrive to the surface environment sparsely crystalline and only modestly differentiated. The subaqueously erupted Serreta basalt is an example of such a magma. If its ^210^Pb deficit resulted from a steady loss of Rn in a CO_2_-rich gas phase, then its (^210^Pb/^226^Ra)_0_ ratio of 0.71 suggests a rise time from the depth of CO_2_-saturation to the surface of 11 years assuming that Rn degassed with perfect efficiency^5^. The required maximum average magma velocity would depend on the initial CO_2_ content of the magma. Assuming 1 wt.% H_2_O, 0.5 wt.% CO_2_ and a crustal density of 3000 kg/m^3^, saturation of the gas phase would occur at about 20 km^69^. The maximum average velocity of Serreta magma in this case would have been about 1.8 km/yr. The (^210^Pb/^226^Ra)_0_ value for the Serreta basalt is nearly identical to the median value for ocean island basalts in general^10^, suggesting that this magnitude of rise time and magma velocity is typical for ocean island basalts in general.

An alternative explanation for ^210^Pb deficits in ocean island basalts and MORB is Ra-Pb fractionation during melting. This explanation is based on weak correlations between (^210^Pb/^226^Ra) and geochemical parameters that vary with changes in degree of melting, such as (^226^Ra/^230^Th)^3^,^10^. However, recent studies of (^210^Pb/^226^Ra) variations in lavas from individual ocean islands^35^,^36^, continental rifts^44^,^45^, and mid-ocean ridges^44^ have not supported melting as the primary cause of (^210^Pb/^226^Ra) variations. These studies have illustrated, for example, that although mineral-melt partitioning during magma generation should only produce ^210^Pb deficits, some lavas from all tectonic settings have ^210^Pb excesses (Fig. 5). Such excesses can result from crystal fractionation of K-feldspar and amphibole, but only in highly differentiated magmas such as phonolite^8^ (Fig. 5), not in magmas that crystallize olivine, augite, and plagioclase like the Serreta basalt^19^.

Only rarely does shallow degassing of water from an ocean island magma cause it to stall out in shallow magma reservoirs for periods of time long enough to equilibrate (^210^Pb/^226^Ra) values. Portions of Iceland offer a physical setting where such magma stagnation and differentiation is common because of its rather water rich parental magmas, glaciation, thicker and somewhat older crust^70^. The benmoreite from Eyjafjallajökull appears to have stagnated at a depth in the crust of about 1.7–5.0 km^71^ without persistent Rn degassing as evidenced by its equilibrium (^210^Pb/^226^Ra) ratio.

As is the case for ocean island basalts, primitive arc magmas must typically begin to saturate in a CO_2_-rich volatile phase at deep crust or mantle depths. The rare arc basalts that erupt with ^210^Pb deficits^10^ reflect this process. However, most arc magmas stall in the crust and differentiate significantly. For parental magmas with 2–6 wt.% water, open-system degassing of water-rich vapor phases begins at depths of 1.5–12 km, which match the observed storage depths for magmas in volcanic arcs^60^. The Yasur lava is likely at the shallow end of this range based on the probable lower water contents^25^ for its parental magmas compared with those from Tonga.

Once arc magmas stall, they must typically maintain (^222^Rn/^226^Ra) equilibrium for most of the time spent in the reservoir system, which means these magmas must not undergo persistent open-system degassing. Pulses of ^222^Rn loss by degassing as magmas resided in the crust are allowed, so long as these pulses were spaced in time long enough to prevent measurable decay of ^210^Pb. Numerical modeling of steady-state systems undergoing periods of complete ^222^Rn loss followed by periods of repose with (^222^Rn) = (^226^Ra) indicates that such systems will have: (^210^Pb/^226^Ra) = 1/(1 + t_d_/t_r_) where t_d_ is the time the system spends degassing and t_r_ is the time spent in repose. In this circumstance, values of (^210^Pb/^226^Ra) remain within analytical error of equilibrium only if t_d_/t_r_ is less than about 0.03, indicating that any magma with equilibrium (^210^Pb/^226^Ra) must not lose Rn for the vast majority of its residence time in the crust.

An alternative explanation is that the fluxes of Rn in and out of the magma are equal. Of these two scenarios, we favor the former because a fortuitous balance between ^222^Rn lost and gained seems unlikely to have occurred in multiple systems. Another alternative is that that ^222^Rn supplied from recharge magmas mutes any ^210^Pb deficit that exists in a resident magma due to degassing. However, this explanation requires magma renewal rates approaching 1 magma volume/year^5^, which basically just explains why equilibrium (^210^Pb/^226^Ra) values are maintained in magmas as they flow rapidly through conduits to the surface. Thus, we conclude that the near equilibrium (^210^Pb/^226^Ra) values for eruptives from Yasur, Hunga Ha’apai, Home Reef, Havre, Fonualei and Eyjafjallajökull require that their parental magmas stalled and differentiated in the crust without persistent open-system degassing for more than a century before eruption. For all of these systems, any open-system loss of ^222^Rn to a gas phase during the final rise toward the surface must have occurred over a period of less than two years to prevent (^210^Pb) from being measurably less than (^226^Ra).

## Conclusions

Parental magmas in arc and ocean island settings typically have concentrations of 0.3–1.3 wt.% CO_2_, which leads to the onset of degassing of magmas in the upper mantle or deep crust. This degassing leads to a steady rise of these magmas and a persistent Rn loss that results in the common ^210^Pb deficits in ocean island settings. The Serreta basalt analyzed here has a near-median (^210^Pb/^226^Ra)_0_ value for an ocean island basalt implying a minimum of 8.5 years of rise to the surface at a velocity as high as about 2.4 km/year after it first saturates in a gas phase. For ocean island basalts, significant H_2_O degassing typically occurs too shallowly to significantly impede their progress toward the surface. An exception appears to be Eyjafjallajökull, where magmas ponded and differentiated to trachytic to rhyolitic compositions within the relatively thick and glaciated crust. Its system of magma chambers must be relatively complex, because the equilibrium (^210^Pb/^226^Ra)_0_ value for the benmoreite suggests that basaltic and silicic endmembers mixed more than a century before it erupted.

Arc magmas typically have significant subducted H_2_O^58^,^60^, and open-system degassing of this water in the middle to upper crust typically will cause magmas to freeze and stall^65^. The commonplace equilibrium (^210^Pb/^226^Ra) values in arc magmas suggest that once these magmas stall, they must not persistently lose or gain Rn for long enough periods or at high enough rates to affect (^210^Pb) values. The further implication is that water-saturated magmas do not generally undergo persistent open-system degassing. Remobilization of these stalled magmas might occur by melt-crystal separation or by pressurization caused by magma recharge^72^.

Water-rich magmas must rise to the surface while degassing over periods of less than 2 years from their final staging reservoir to maintain (^210^Pb/^226^Ra) values within analytical error of secular equilibrium. Rise times for water-saturated magmas between staging reservoirs could be similarly rapid. In this circumstance, equilibrium (^210^Pb/^226^Ra) values will be maintained throughout magma systems that are saturated in a water-rich volatile phase. Quiescent final staging reservoirs and rapid rise times to the surface implied by equilibrium (^210^Pb/^226^Ra) values in water-laden magmas suggests an inherent lack of long-term predictability of explosive eruptions.

## Methods

### Major elements

Hand specimens were crushed using a stainless steel jaw press and the chips were then ultrasonically washed in MilliQ H_2_O to remove any seawater contamination. The Havre rhyolite was washed with weak HCl and water. The washed chips were subsequently dried and then powdered in an agate mill. Major element concentrations were determined on a Siemens^®^ SRS300 XRF at the University of Auckland, following standard techniques^73^. Fresh glass was analyzed for the Havre rhyolite. A polished thin section was carbon coated and analyzed by a Cameca SX100 electron microprobe at Macquarie University. A defocused beam was used with an accelerating voltage of 15 kV and a beam current of 15 nA. Counting times of 10 s were used for both peak and background measurements. The analysis in Table 1 represents the average of 5 spots. Spectrometer calibration was achieved using the following standards: Jadeite (Na), Fayalite (Fe), kyanite (Al), olivine (Mg), chromite (Cr), spessartine garnet (Mn), orthoclase (K), wollastonite (Ca, Si) and TiO_2_ (Ti).

### Trace elements

Sample powders were dissolved in a HF-HNO_3_ mixture for trace element analysis. Most samples were analyzed at Macquarie University using an Agilent^®^ 7500CS ICP-MS following procedures outlined in Eggins *et al*.^74^. Detection limits during the period of study are listed in the Supplemental Materials Table S2. Sample EJ-1 was analyzed using a Thermo X-series II ICP-MS at the University of Iowa using methods described in Peate *et al*.^75^.

### Radiogenic isotopes

Sr-Nd isotope analysis was performed on ~100 mg of powdered sample that was digested in a HF-HNO_3_ mix in heated Teflon beakers. Sr and REE fractions were separated using a cationic column containing Biorad^®^ AG50W-X8 (200–400 mesh) cationic exchange resin, after which Sm and Nd were separated using EIChrom^®^ LN-spec resin following the column procedure given by Pin *et al*.^76^. Samples were loaded on to out-gassed single (Sr) and double (Nd) rhenium filaments using 2 μl of TaCl_5_ + HF + H_3_PO_4_ + H_2_O_2_ and 5 μl of 1 N HCl, 0.35 N H_3_PO_4_ activator solutions, respectively. Analyses were performed in static mode on a ThermoFinnigan Triton^®^ TIMS at Macquarie University. Instrument mass fractionation was accounted for by normalizing ^87^Sr/^86^Sr and ^143^Nd/^144^Nd to ^87^Sr/^86^Sr = 0.1194 and ^143^Nd/^144^Nd = 0.7219, respectively. Analysis of SRM-987 yielded an ^87^Sr/^86^Sr value of 0.710272 ± 4. Values for USGS rock standard BHVO-2 are listed in Supplementary Information Table S2. Sr and Nd blanks were lower than 1000 and 80 pg, respectively. No corrections for blanks were made. Repeated measurements of standard materials during the data acquisition period yielded the following values: ^87^Sr/^86^Sr of 0.710259 ± 0.000038 (2 SD; n = 42) for the SRM-987 standard; ^87^Sr/^86^Sr of 0.703493 ± 0.000068 (2 SD; n = 52) for BHVO-1; ^143^Nd/^144^Nd of 0.511112 ± 0.000017 (2 S.D.; n = 32) for the JMC Nd (321) standard and ^143^Nd/^144^Nd of 0.512971 ± 0.000025 (2 SD; n = 45) for BHVO-1.

### U-series

Samples were spiked with ^236^U-^229^Th and ^228^Ra tracers and dissolved using an HF-HNO_3_-HCl mix in heated teflon pressure bombs. Samples were brought into solution, dried, and U, Th and Ra were separated chromatographically^76^. U and Th concentrations and isotope ratios were measured in dynamic mode on a Nu Instruments^®^ MC-ICP-MS at Macquarie University. Mass bias was determined assuming ^238^U/^235^U = 137.88 and the IC0 gain was determined during interspersed dynamic analyses of CRM145 assuming a ^234^U/^238^U ratio of 5.286 × 10^−5 ^ 77,78. Radium aliquots were loaded onto degassed Re filaments using a Ta-HF-H_3_PO_4_ activator solution^78^ and ^228^Ra/^226^Ra ratios were measured to a precision typically ~0.5% in dynamic ion counting mode on the TIMS at Macquarie University. Most of the samples were analyzed with TML and BCR-2 standards^79^,^80^ between 2008 and 2011 (see Supplementary Information Table S2). For the Serreta and Havre Ra analyses that were performed in 2015, the ^228^Ra concentration of the spike was determined at the same time as the unknowns by analyzing the TML rock standard and assuming secular equilibrium. Analysis of BCR-2 performed at the same time yielded values that were within 3% of secular equilibrium (see Supplementary Information Table S2). Estimated total analytical errors (2σ) for activity ratios listed in Table 2 include errors on the precision of each analysis and the uncertainties of spike values based on replicate analyses of standards.

For the ^210^Pb data, 1–2 g aliquots of sample powder were leached in 0.25 N HCl for 20 minutes before decanting the supernatant solution. The residual powder was then rinsed 3 times in MilliQ H_2_O before drying. The leachates and the leached powders were then spiked with ^209^Po and analysed by α-counting on an EGG Ortec^®^ system at the University of Iowa in 2010 and 2011^12^,^13^,^43^. The (^210^Pb/^226^Ra)_o_ values used in Table 1 are based on (^210^Po) and assume secular equilibrium between ^210^Pb and ^210^Po (t_1/2_ = 138 days) for samples analyzed more than 2 years after eruption. For samples Van-A1 and HH09-01, multiple measurements of ^210^Po in-growth over about 3 years allowed for extrapolation to equilibrium (^210^Pb)_o_ values (see Supplementary Information Table S1). Errors quoted in Table 2 are 2σ. (^210^Po) values measured for USGS standards BCR- 2 and RGM-2 during sample analysis are in Supplementary Information Table S2.

## Additional Information

**How to cite this article:** Reagan, M. *et al*.^210^Pb-^226^Ra disequilibria in young gas-laden magmas. *Sci. Rep.* **7**, 45186; doi: 10.1038/srep45186 (2017).

**Publisher's note:** Springer Nature remains neutral with regard to jurisdictional claims in published maps and institutional affiliations.

## Supplementary Material

Supplementary Materials Table 1

Supplementary Materials Table 2

## Figures and Tables

**Table 1:** Major and trace element compositions of young volcanic rocks.

Volcano	Yasur	Hunga	Hunga	Home Reef	Fonualei	Havre	Terceira	Eyjafjallajökull
Province	Vanuatu	Tonga	Tonga	Tonga	Tonga	Kermadec	Azores	Iceland
Sample	Van A1	HH09-01	HH09-02	HR06	F0805	PC	Serreta	EJ-1
Month-yr	08–2008	04–2009	04–2009	11–2006	1979–90^a^	07–2012	02–1999	04–2010
SiO_2_	55.53	57.56	57.96	64.45	66.03	72.70	47.27	56.62
TiO_2_	0.70	0.63	0.62	0.70	0.59	0.38	2.76	1.72
Al_2_O_3_	18.38	14.34	14.36	13.55	13.51	13.44	14.85	14.95
Fe_2_O_3_	7.92	11.66	11.67	8.94	8.67	2.79	11.34	10.65
MnO	0.14	0.18	0.18	0.17	0.19	0.11	0.15	0.24
MgO	2.77	3.51	3.55	1.80	1.41	0.43	8.57	2.23
CaO	7.44	8.76	8.77	6.27	5.56	2.14	10.43	5.37
Na_2_O	3.84	2.04	2.05	3.13	3.07	5.59	3.10	5.24
K_2_O	2.55	0.54	0.53	0.83	1.19	1.60	0.70	1.74
P_2_O_5_	0.41	0.09	0.10	0.21	0.27	0.06	0.39	0.48
Total	99.68	99.32	99.79	100.05	100.49	99.24	99.56	99.24
Li (ppm)	10.2	6.6	6.6	—	8.1	13.0	4.4	14.1
Be	1.3	0.3	0.3	—	0.7	1.1	1.4	3.3
Sc	18	47	46	30	29	13	27	16
V	222	400	395	131	73	21	235	81
Cr	20	9	11	5	3	0	276	20
Co	20	31	31	16	13	4	46	14
Ni	21	18	18	6	1	0	170	15
Cu	282	153	150	32	28	8	51	26
Zn	78	89	88	156	108	68	100	133
Rb	35.3	6.3	6.2	14.6	16.0	26.8	16.2	36.1
Sr	660	174	172	254	293	153	476	341
Y	24.0	19.3	19.1	22.2	25.0	45.6	29.3	62.1
Zr	96.9	30.5	30.1	42	47.8	174	197.6	447.8
Nb	1.31	0.28	0.27	0.60	0.89	2.20	33.7	57.1
Cs	1.24	0.39	0.40	0.61	0.612	1.26	0.16	0.47
Ba	506	145	150	296	255	422	185	403
La	11.6	1.54	1.60	4.65	4.580	12.10	21.2	49.37
Ce	27.3	4.24	4.39	19.64	11.14	29.90	45.9	109.8
Pr	3.88	0.73	0.76	2.30	1.81	4.28	6.10	14.05
Nd	17.3	3.79	3.94	9.93	8.88	20.60	26.12	58.89
Sm	4.22	1.37	1.44	2.93	2.82	5.58	6.18	13.32
Eu	1.31	0.50	0.53	1.07	0.94	1.53	2.05	4.34
Gd	4.11	1.75	1.86	3.38	3.33	6.22	6.27	12.95
Tb	0.62	0.34	0.36	0.56	0.60	1.06	0.94	2.01
Dy	3.67	2.35	2.48	4.01	3.76	6.98	5.19	11.63
Ho	0.79	0.54	0.58	0.86	0.86	1.52	0.98	2.26
Er	2.27	1.63	1.74	2.51	2.57	4.59	2.52	6.09
Tm	—	—	—	0.38	—	0.71	—	0.85
Yb	2.20	1.63	1.74	2.75	2.54	4.78	1.95	5.40
Lu	0.34	0.25	0.27	0.40	0.402	0.730	0.28	0.77
Hf	2.61	0.76	0.83	1.39	1.464	4.660	4.24	10.84
Ta	0.04	0.03	0.02	0.07	0.03	0.17	1.93	3.89
Pb	12.60	1.60	1.76	5.82	4.40	5.97	1.35	3.90
Th	1.828	0.129	0.142	0.236	0.440	2.292	2.114	5.049
U	1.057	0.133	0.140	0.203	0.370	0.752	0.818	1.675

**Table 2:** Isotope and U-series data.

Volcano	Yasur	Hunga	Hunga	Home Reef	Fonualei	Havre	Terceira	Eyjafjallajökull
Province	Vanuatu	Tonga	Tonga	Tonga	Tonga	Kermadec	Azores	Iceland
Sample	Van A1	HH09-01	HH09-02	HR06	F0805	PC	Serreta	EJ-1
Month-yr	08–2008	04–2009	04–2009	11–2006	1979–90^a^	07–2012	02–1996	04–2010
^87^Sr/^86^Sr	0.703540	0.703951	0.703688	0.703636	0.703769	0.703705	0.703533	0.703257
±2σ	0.0000072	0.0000062	0.0000096	0.0000064	0.0000063	0.0000062	0.0000075	0.0000068
^143^Nd/^144^Nd	0.513030	0.513028	0.513026	0.512987	0.512948	0.513115	0.512925	0.513004
±2σ	0.0000038	0.0000048	0.0000038	0.0000012	0.0000025	0.0000035	0.0000036	0.0000042
^226^Ra fg/g	505.3	115.7	115.9	185.8	236.0	326.7	452.3	654.1
±2σ	13.1	2.9	2.9	5.0	6.2	8.3	12.6	16.4
(^210^Pb) dpm/g	1.122*	0.264*	0.262	0.457	0.510	0.695	0.796	1.435
±2σ	0.022	0.010	0.018	0.030	0.019	0.026	0.026	0.038
(^234^U/^238^U)	0.995	0.999	0.991	0.996	1.016	1.005	1.006	1.005
±2σ	0.005	0.006	0.005	0.006	0.005	0.005	0.005	0.005
(^238^U/^232^Th)	1.754	3.128	2.991	2.610	2.551	0.996	1.174	1.007
±2σ	0.030	0.053	0.051	0.044	0.044	0.017	0.020	0.017
(^230^Th/^232^Th)	1.459	1.679	1.700	1.611	1.656	0.930	1.399	1.092
±2σ	0.015	0.040	0.050	0.023	0.017	0.009	0.014	0.011
(^230^Th/^238^U)	0.831	0.539	0.568	0.617	0.649	0.934	1.192	1.085
±2σ	0.015	0.014	0.018	0.010	0.010	0.016	0.020	0.019
(^226^Ra/^230^Th)	1.703	4.796	4.316	4.392	2.911	1.377	1.374	1.066
±2σ	0.049	0.134	0.120	0.129	0.084	0.038	0.042	0.030
(^210^Pb/^226^Ra)	1.012	1.040	1.022	1.121	0.985	0.970	0.802	1.000
(^210^Pb/^226^Ra)_0_	1.012	1.040	1.025	1.144	0.966	0.968	0.711	1.000
±2σ	0.033	0.047	0.080	0.097	0.119	0.047	0.055	0.038

^a^For the calculation of (^210^Pb/^226^Ra)_0_ for sample F0805 we adopted an age of 1/1985.

^*^Value determined by regression through multiple (^210^Po) analyses (see Supplementary Table 1).

## References

[b1] Bourdon B., Turner S. & Dosseto A. Dehydration and partial melting in subduction zones: Constraints from U-series disequilibria. J. Geophys. Res. 108, 2291, doi: 10.1029/2002JB001839 (2003).

[b2] Peate D. W. & Hawkesworth C. J. U. series disequilibria; insights into mantle melting and the timescales of magma differentiation. Rev. Geophys. 43, 1–43 RG1003, doi: 10.1029/2004RG000154 (2005).

[b3] Rubin K. H., van der Zander I., Smith M. C. & Bergmanis E. C. Minimum speed limit for ocean ridge magmatism from ^210^Pb-^226^Ra-^230^Th disequilibria. Nature 437, 534–538 (2005). 16177787 10.1038/nature03993

[b4] Dosseto A., Turner S. P. & Van Orman J. A. Timescales of Magmatic Processes: From Core to Atmosphere. Wiley-Blackwell. 264 pp (2010).

[b5] Gauthier P.-J. & Condomines M. ^210^Pb-^226^Ra radioactive disequilibria in recent lavas and radon degassing: inferences on the magma chamber dynamics at Stromboli and Merapi volcanoes. Earth Planet. Sci. Lett. 172, 111–126 (1999).

[b6] Williams R. W., Gill J. B. & Bruland K. W. Ra-Th disequilibria systematics: timescales of carbonatite magma formation at Oldoinyo Lengai volcano, Tanzania. Geochim. Cosmochim. Acta 50, 1249–1259 (1986).

[b7] Turner S., Black S. & Berlo K. ^210^Pb-^226^Ra and ^232^Th-^228^Ra systematics in young arc lavas: implications for magma degassing and ascent rates. Earth Planet. Sci. Lett. 227, 1–16 (2004).

[b8] Reagan M. K., Turner S., Legg M., Sims K. W. W. & Hards V. L. ^238^U- and ^232^Th-decay series constraints on the timescales of crystal fractionation to produce the phonolite erupted in 2004 near Tristan da Cunha, South Atlantic Ocean. Geochim. Cosmochim. Acta 72, 4367–4378 (2008).

[b9] Sims K. W. W. . On the timescales of magma genesis, melt evolution, crystal growth rates and magma degassing in the Erebus volcano magmatic system using the ^238^U-, ^235^U- and ^232^Th-decay series. J. Petrol 54, 235–271 (2013).

[b10] Berlo K. & Turner S. Origins of ^210^Pb-^226^Ra disequilibria in young volcanic rocks. Earth Planet. Sci. Lett. 296, 155–164 (2010).

[b11] Berlo K., Turner S., Blundy J. Black S. & Hawkesworth C. Tracing pre-eruptive magma degassing using (^210^Pb/^226^Ra) disequilibria in the volcanic deposits of the 1980–1986 eruption of Mount St. Helens Earth Planet. Sci. Lett. 249, 337–349 (2006).

[b12] Reagan M. K., Tepley F. J., Gill J. B., Wortel M. & Hartman B. Rapid time scales of basalt to andesite differentiation at Anatahan volcano, Mariana Islands. J. Volcanol. Geotherm. Res. 146, 171–183 (2005).

[b13] Reagan M. K., Tepley F. J. III, Gill J. B., Wortel M. & Garrison J. Timescales of degassing and crystallization implied by ^210^Po-^210^Pb-^226^Ra disequilibria for andesitic lavas erupted from Arenal volcano. J. Volcanol. Geotherm. Res. 157, 135–146 (2006).

[b14] Kayzar T. M., Cooper K. M., Reagan M. K. & Kent A. J. R. Gas transport model for the magmatic system at Mount Pinatubo, Philippines: Insights from (^210^Pb)/(^226^Ra). J. Volcanol. Geotherm. Res. 181, 124–140 (2009).

[b15] Turner S. . Magma evolution in the primitive, intra-oceanic Tonga arc: Rapid petrogenesis of dacites at Fonualei volcano. J. Petrol. 53, 1231–1253 (2012).

[b16] Carey R. J., Wysoczanski R., Wunderman R. & Jutzeler M. Discovery of the largest historic silicic submarine eruption. EOS 95, 157–164 (2014).

[b17] Jutzeler M. . On the fate of pumice rafts formed during the 2012 Havre submarine eruption. Nature Comm.doi: 10.1038/ncomms4660 (2014). PMC3997806 24755668

[b18] Kremers S. . Shallow magma-mingling-driven Strombolian eruptions at Mt. Yasur volcano, Vanuatu. Geophys. Res. Lett. 39, L21304, doi: 10.1029/2012gl053312 (2012).

[b19] Kueppers U., Nichols A. R. J., Zanon V., Potuzak M. & Pacheco J. M. R. Lava balloons – peculiar products of basaltic submarine eruptions. Bull. Volcanol. 74, 1379–1393 (2012).

[b20] Gislason S. R., Alfredsson H. A., Eiriksdottir E. S., Hassenkam T. & Stipp S. L. S. Volcanic ash from the 2010 Eyjafjallajökull eruption. Applied Geochem. 26, S188–S190 (2011).

[b21] Sigmarsson O. . Remobilization of silicic intrusion by mafic magmas during the 2010 Eyjafjallajökull eruption. Solid Earth 2, 271–281 (2011).

[b22] Sigmarsson O., Condomines M. & Gauthier P.-J. Excess ^210^Po in 2010 Eyjafjallajökull tephra (Iceland): Evidence for pre-eruptive gas accumulation. Earth Planet. Sci. Lett. 427, 66–73 (2015).

[b23] Cioni R. . Insights into the dynamics and evolution of the 2010 Eyjafjallajökull summit eruption (Iceland) provided by volcanic ash textures. Earth Planet. Sci. Lett. 394, 111–123 (2014).

[b24] Borisova A. Y., Toutain J.-P., Stefansson A., Gouy S. & de Parseval1 P. Processes controlling the 2010 Eyjafjallajökull explosive eruption. J. Geophys. Res. 117, B05202, doi: 10.1029/2012JB009213 (2012).

[b25] Métrich N. . Magma and volatile supply to post-collapse volcanism and block resurgence in Siwi Caldera (Tanna Island,Vanuatu Arc). J. Petrol. 52, 1077–1105 (2011).

[b26] Firth C. W., Handley H. K., Cronin S. J. & Turner S. P. The eruptive history and chemical stratigraphy of a post-caldera, steady-state volcano: Yasur, Vanuatu. bull. Volcanol. 76, 837 (2014).

[b27] Rotella M. D. . Dynamics of deep submarine silicic explosive eruptions in the Kermadec arc, as reflected in pumice vesicularity textures. J. Volcanol. Geotherm. Res. 301, 314–332 (2015).

[b28] Turner S. . ^238^U-^230^Th disequilibria, magma petrogenesis and flux rates beneath the depleted Tonga-Kermadec island arc. Geochim. Cosmochim. Acta 61, 4855–4884 (1997).

[b29] Turner S. P., Peate D. W., Hawkesworth C. J., Eggins S. M. & Crawford A. J. Two mantle domains and the time scales of fluid transfer beneath the Vanuatu arc. Geology 27, 963–966 (1999).

[b30] Widom E., Carlson R. W., Gill J. B. & Schmincke H.-U. Th–Sr–Nd–Pb isotope and trace element evidence for the origin of the São Miguel, Azores, enriched mantle source. Chem. Geol. 140, 49–68 (1997).

[b31] Turner S., Hawkesworth C., Rogers N. & King P. U-Th disequilibria and ocean island basalt generation in the Azores. Chem. Geol. 139, 145–164 (1997).

[b32] Turner S., Bourdon B., Hawkesworth C. & Evans P. ^226^Ra-^230^Th evidence for multiple dehydration events, rapid melt ascent and the time scales of differentiation beneath the Tonga-Kermadec island arc. Earth Planet. Sci. Lett. 179, 581–593 (2000).

[b33] Turner S., Evans P. & Hawkesworth C. Ultra-fast source to-surface movement of melt at island arcs from ^226^Ra-^230^Th systematics. Science 292, 1363–1366 (2001). 11359009 10.1126/science.1059904

[b34] Sims K. W. W. & Hart S. R. Comparison of Th, Sr, Nd and Pb isotopes in oceanic basalts: Implications for mantle heterogeneity and magma genesis. Earth Planet. Sci. Lett. 245, 742–761 (2006).

[b35] Sigmarsson O. & Condomines M. Bachèlery Magma residence time beneath the Piton de la Fournaise Volcano, Reunion Island, from U-series disequilibria. Earth Planet. Sci. Lett. 234, 223–134 (2005).

[b36] Girard G. . ^238^U-^230^Th-^226^Ra-^210^Pb-^210^Po disequilibria constraints on magma generation, ascent, and degassing during the ongoing eruption of Kīlauea. J. Petrol.(submitted).

[b37] Reagan M. K., Cooper K. M., Pallister J. S., Thornber C. R. & Wortel M. Timing of degassing and plagioclase growth in lavas erupted from Mount St. Helens, 2004–2005, from ^210^Po-^210^Pb-^226^Ra disequilibria, *in* Sherrod, D. R., Scott, W. E., and Stauffer, P. H., A volcano rekindled: the first year of renewed eruption at Mount St. Helens, 2004–2006. US Geol. Surv. Prof. Pap. 1750, 847–856 (2008).

[b38] Syracuse E. M., van Keken P. E. & Abers G. A. The global range of subduction zone thermal models. Phys. Earth Planet. Interiors 183, 73–90 (2010).

[b39] Plank T., Cooper L. B. & Manning C. E. Emerging geothermometers for estimating slab surface temperatures. Nature Geosci. 2, 611–615 (2009).

[b40] Plank T. & Langmuir C. H. The chemical composition of subducting sediment and its consequences for the crust and mantle. Chem. Geol. 145, 325–394 (1998).

[b41] Kelley K. A., Plank T., Ludden J. & Staudigel H. Composition of altered oceanic crust at ODP Sites 801 and 1149. Geochem. Geophys. Geosys. 4, doi: 10.1029/2002GC00043 (2003).

[b42] Jenner F. E. & O’Neill H. St.C. Analysis of 60 elements in 616 ocean floor basaltic glasses. Geochem. Geophys. Geosys. 13, doi: 10.1029/2011GC004009 (2012).

[b43] Waters C. L. . Sill to surface: Linking young off-axis volcanism with subsurface melt at the overlapping spreading center at 9^o^03′N East Pacific Rise. Earth Planet. Sci. Lett. 369–370, 59–70 (2013).

[b44] Turner S., Reagan M., Vigier N. & Bourdon B. Origin of ^210^Pb-^226^Ra disequilibria in basalts - new insights from the 1978 Asal Rift eruption. Geochem. Geophys. Geosys. 13, doi: 10.1029/2012GC004173 (2012).

[b45] Turner M. B. . Timescales of magma degassing – insights from U-series disequilibria, Mount Cameroon, West Africa. J. Volcanol. Geotherm. Res. 262, 38–46 (2013).

[b46] Sigmarsson O. Short magma residence time at an Icelandic volcano inferred from U-series disequilibria. Nature 382, 440–442 (1996).

[b47] Handley H. K., Turner S., Berlo K., Beier C. & Saal A. Insights into the Galapagos plume from Uranium-series isotopes of recently erupted basalts. Geochem. Geophys. Geosyst.doi: 10.1029/2011GC003676 (2011).

[b48] Condomines M., Sigmarsson O. & Gauthier P.-J. A simple model of ^222^Rn accumulation leading to ^210^Pb excesses in volcanic rocks. Earth Planet. Sci. Lett. 293, 331–338 (2010).

[b49] Bourdon B., Turner S. & Ribe N. M. Partial melting and upwelling rates beneath the Azores from a U-series isotope perspective. Earth Planet. Sci. Lett. 239, 42–56 (2005).

[b50] Bourdon B., Ribe N. M., Stracke A., Saal A. E. & Turner S. P. Insights into the dynamics of mantle plumes from uranium-series geochemistry. Nature 444, 713–717 (2006). 17151659 10.1038/nature05341

[b51] Stracke A., Bourdon B. & McKenzie D. Melt extraction in the Earth’s mantle: constraints from U-Th-Pa-Ra studies in oceanic basalts. Earth Planet. Sci. Lett. 244, 97–112 (2006).

[b52] Beier C. . Geochemical evidence for melting of carbonated peridotite on Santa Maria Island, Azores. Contrib. Mineral. Petrol. 165, 823–841 (2013).

[b53] Gill J. B., Williams R. W. & Bruland K. Eruption of basalt and andesite lava degasses ^222^Rn and ^210^Po. Geophys. Res. Lett. 12, 17–20 (1985).

[b54] Burton M. R., Sawyer G. M. & Granieri D. Deep Carbon Emissions from Volcanoes. Rev. Mineral. & Geochem. 75 323–354 (2013).

[b55] Métrich N. & Wallace P. J. Volatile abundances in basaltic magmas and their degassing paths tracked by melt inclusions. Rev. Mineral. Geochem. 69, 363–402 (2008).

[b56] Moune S., Sigmarsson O., Schiano P., Thordarson T. & Keiding J. K. Melt inclusion constraints on the magma source of Eyjafjallajökull 2010 flank eruption. J. Geophys. Res. 117, doi: 10.1029/2011JB008718 (2012).

[b57] Hartley M. E., Maclennan J., Edmonds M. & Thordarson T. Reconstructing the deep CO_2_ degassing behaviour of large basaltic fissure eruptions. Earth Planet. Sci. Lett. 393, 120–131 (2014).

[b58] Wallace P. J. Volatiles in subduction zone magmas: concentrations and fluxes based on melt inclusion and volcanic gas data. J. Volcanol. Geotherm. Res. 140, 217–240 (2005).

[b59] Caulfield J. T., Turner S. P., Smith I. E. M., Cooper L. B. & Jenner G. A. Magma evolution in the primitive, intra-oceanic Tonga arc: Petrogenesis of basaltic andesites at Tofua Volcano. J. Petrol. 53, 1197–1230 (2012).

[b60] Plank T., Kelley K. A., Zimmer M. M., Hauri E. H. & Wallace P. J. Why do mafic arc magmas contain ~4 wt% water on average? Earth Planet. Sci. Lett. 364, 168–179 (2013).

[b61] Morizet Y., Nichols A. R. L., Kohn S. C., Brooker R. A. & Dingwell D. B. The influence of H_2_O and CO_2_ on the glass transition temperature: insights into the effects of volatiles on magma viscosity. Eur. J. Mineral. 19, 657–669 (2007).

[b62] Seifert R., Malfait W. J., Lerch P. & Sanchez-Valle C. Partial molar volume and compressibility of dissolved CO_2_ in glasses with magmatic compositions. Chem. Geol. 358, 119–130 (2013).

[b63] Dixon J. E. & Stolper E. M. An experimental study of water and carbon dioxide solubilities in mid-ocean ridge basaltic liquids. part II: applications to degassing. J. Petrol. 36, 1633–1646 (1995).

[b64] Blundy J., Cashman K. & Humphreys M. Magma heating by decompression-driven crystallization beneath andesite volcanoes. Nature 443, 76–80 (2006). 16957729 10.1038/nature05100

[b65] Sparks R. S. J. Dynamics of magma degassing. Geol. Soc. London Spec. Pub. 213, 5–22 (2003).

[b66] Blundy J. & Cashman K. Rapid decompression-driven crystallization recorded by melt inclusions from Mount St. Helens volcano 2005. Geology 33, 793–796 (2005).

[b67] Llewellin E. W. & Manga M. Bubble suspension rheology and implications for conduit flow. J. Volcanol. Geotherm. Res. 143, 205–217 (2005).

[b68] Cashman K. & Sparks R. J. S. How volcanoes work: A 25 year perspective. Geol. Soc. Am. Bull. 125, 664–690 (2013).

[b69] Papale P., Moretti R. & Barbato D. The compositional dependence of the saturation surface of H_2_O + CO_2_ fluids in silicate melts. Chem. Geol. 229, 78–95 (2006).

[b70] Thirlwall M. F., Gee M. A. M., Lowry D., Mattey D. P., Murton B. J. & Taylor R. N. Low δ^18^O in the Icelandic mantle and its origins: Evidence from Reykjanes Ridge and Icelandic lavas. Geochim. Cosmochim. Acta 70, 993–1019 (2006).

[b71] Keiding J. K. & Sigmarsson O. Geothermobarometry of the 2010 Eyjafjallajökull eruption: New constraints on Icelandic magma plumbing systems. J. Geophys. Res. 117, doi: 10.1029/2011JB008829 (2012).

[b72] Sparks R. S. J., Sigurdsson H. & Wilson L. Magma mixing: a mechanism for triggering acid explosive eruptions. Nature 267,315–318 (1977).

[b73] Norrish K. & Hutton J. T. An accurate x-ray spectrograph method for the analysis of a wide range of geological samples. Geochim. Cosmochim. Acta 33, 431–453 (1969).

[b74] Eggins S. . A simple method for the precise determination of >40 trace elements in geological samples by ICPMS using enriched isotope internal standardisation. Chem. Geol. 134, 311–326 (1997).

[b75] Peate D. W. . Compositional characteristics and spatial distribution of enriched Icelandic mantle components. J. Petrol. 51, 1447–1475 (2010).

[b76] Pin C. & Zalduegui J. F. S. Sequential separation of light rare-earth elements, thorium and uranium by miniaturized extraction chromatography: Application to isotopic analyses of silicate rocks. Analytica Chimica Acta 339, 79–89 (1997).

[b77] Turner S., Beier C., Niu Y. & Cook C. U-Th-Ra disequilibria and the extent of off-axis volcanism across the East Pacific Rise at 9°30′N, 10°30′N and 11°20′N. Geochem. Geophys. Geosys, doi: 10.1029/2010GC003403 (2011).

[b78] Cheng H. . The half lives of uranium-234 and thorium-230. Chem. Geol. 169, 17–33 (2000).

[b79] Birck J. L. Precision K-Rb-Sr isotope analysis-application to Rb-Sr chronology. Chemical Geology 56, 73–83 (1986).

[b80] Sims K. W. W. . An inter-laboratory assessment of the Th isotopic composition of synthetic and rock standards. Geostandards and Analytical Research 32, 65–91 (2008).

