# Supplementary material for: 210Pb-226Ra disequilibria in young gas-laden magmas
Source: Sci Rep. 2017 Mar 24;7:45186. doi: 10.1038/srep45186 (PMC5364531; doi:10.1038/srep45186)
Supplement: Supplementary Materials Table 1 [file srep45186-s1.pdf]

Table S1 ^{210}Po Analyses (listed errors are 2σ)

Location	Yasur	Hunga	Hunga	Home Reef	Fonualei	Havre	Terceira	Eyjafjallajökull
Sample	VAN A1	HH09-01	HH09-02	HR06	F0805	PC	SERRETA	EJ-1
Eruption mo-yr	Aug-08	Apr-09	Apr-09	Nov-06	1979-90a	Jul-12	Feb-96	Apr-10
date of analysis	6/13/09	6/13/09	5/23/12	3/21/10	7/15/11	6/4/14	6/14/11	6/14/11
(^{210}Po) dpm/g	0.904	0.103	0.262	0.457	0.510	0.695	0.796	1.435
±	0.034	0.01	0.018	0.030	0.019	0.026	0.026	0.038
date of analysis	8/21/09	1/8/10						
(^{210}Po) dpm/g	1.09	0.215						
±	0.038	0.016						
date of analysis	3/27/12	2/18/10						
(^{210}Po) dpm/g	1.129	0.214						
±	0.04	0.016						
date of analysis		5/13/12						
(^{210}Po) dpm/g		0.263						
±		0.016						
date of analysis	6/13/09		5/23/12	6/13/09				
leachate (^{210}Po)	3.012		1.512	6.493				
±	0.259		0.247	0.6				
