# Supplementary material for: 210Pb-226Ra disequilibria in young gas-laden magmas
Source: Sci Rep. 2017 Mar 24;7:45186. doi: 10.1038/srep45186 (PMC5364531; doi:10.1038/srep45186)
Supplement: Supplementary Materials Table 2 [file srep45186-s2.pdf]

Table S2. Element abundances and U-series data for standards. Listed errors are 2σ (n=number of analyses).

	Detection limits*	JA1*	BHVO-2*	+/-	TML*	+/-	n	BCR-2*	+/-	n	RGM-2#	+/-	W-2#	+/-
SiO2		64.8												
TiO2		0.84												
Al2O3		15.27												
Fe2O3		7.04												
MnO		0.16												
MgO		1.54												
CaO		5.68												
Na2O		3.87												
K2O		0.78												
P2O5		0.16												
Li	0.005		4.5	0.4									9.4	0.6
Be	0.007		1.38	0.14									0.63	0.06
Sc	0.083		34.6	2.2									35.7	0.5
V	0.035		332.5	21.8									268.2	2.2
Cr	0.109		267.5	23.8									90.3	1.4
Co	0.045		46.1	3.7									43.8	0.3
Ni	0.084		135.1	9.4									70.3	0.7
Cu	0.183		119.5	9.2									101	2.7
Zn	0.157		104.2	7.5									81.3	2.3
Rb	0.073		9.39	0.6									19.77	0.3
Sr	0.392		397	23.1									198	1.2
Y	0.069		29	1.82									21.7	0.08
Zr	0.032		184	14.1									87.7	3.4
Nb	0.008		19.5	1.4									7.5	0.2
Cs	0.0004		0.1	0.01									0.88	0.02
Ba	1.06		132	8.3									171	1.9
La	0.099		15.45	1									10.63	0.1
Ce	0.168		37.55	2.21									23.07	0.21
Pr	0.036		5.42	0.41									2.97	0.04
Nd	0.078		24.48	1.79									12.88	0.11
Sm	0.037		6.18	0.42									3.29	0.04
Eu	0.035		2.01	0.14									1.11	0.02
Gd	0.035		6.21	0.39									3.64	0.02
Tb	0.036		0.96	0.06									0.61	0.01
Dy	0.036		5.25	0.38									3.82	0.03
Ho	0.037		1	0.06									0.78	0.01
Er	0.036		2.52	0.17									2.19	0.01
Tm													0.32	0.01
Yb	0.045		1.96	0.13									2.03	0.03
Lu	0.051		0.27	0.02									0.3	0.01
Hf	0.000		4.32	0.29									2.43	0.13
Ta	0.013		1.12	0.08									0.49	0.04
Pb	0.038		1.59	0.18									7.91	0.34
Th	0.001		1.20	0.10	29.03	0.29	5	5.714	0.114	3				
U	0.009		0.406	0.04	10.32	0.1	5	1.665	0.051	3				
226Ra fg/g					3532	106	5	546	8.4	3				
210Pb dpm/g								1.267#	0.051		4.093#	0.1		
(234U/238U)					1.002	0.005	5	1.000	0.001	3				
(238U/232Th)					1.078	0.015	5	0.884	0.018	3				
(230Th/232Th)					1.082	0.010	5	0.877	0.005	3				
(230Th/238U)					1.004	0.016	5	0.992	0.015	3				
(226Ra/230Th)					1.020	0.032	5	0.975	0.031	3				

* - Macquarie University
\# - University of Iowa
